# Revolutionizing medical education: Surgery takes the lead in virtual reality research

**DOI:** 10.1016/j.sopen.2024.06.013

**Published:** 2024-07-03

**Authors:** M.A. Wolf, M. Mergen, P. Winter, S. Landgraeber, P. Orth

**Affiliations:** a Department of Orthopedics, Saarland University, Kirrberger Straße 100, 66421 Homburg, Saarland, Germany; b Department of Pediatric Oncology and Hematology, Saarland University, Kirrberger Straße 100, 66421 Homburg, Saarland, Germany

**Keywords:** Virtual reality, VR, Medical education, Surgery, Digital

## Abstract

**Objectives:**

Advancements in technology have spurred a transformative shift in medical education, with virtual reality (VR) emerging as a powerful tool for enhancing the learning experience. This study analyses the publications of VR in medical education, focusing on differences within different medical specialties.

**Design:**

Using specific search terms, all studies published on VR in medical education listed in the Web of Science databases were included. All identified publications were analysed in order to draw comparative conclusions regarding their qualitative and quantitative scientific merit.

**Results:**

Since the first publication in 1993 and until the year 2022, there have been 1534 publications on VR in medical education. Over the years, the annual publication rate has increased almost exponentially. The studies have in total been cited 42,655 times (average 27.64 citations/publication). The leading medical field was surgery (415 publications), followed by internal medicine (117 publications), neurology (77 publications) and radiology and nuclear medicine (75 publications). Internationally, the United States (560 publications), the United Kingdom (179 publications), Canada (156 publications), Germany (139 publications) and China (100 publications) are the leading countries in this field. 37.1 % of the publications reported having received funding. Among the 100 organizations with the highest number of grants, only 8 were private companies.

**Conclusion:**

During the last 30 years, there has been a consistent rise in publications, with a notable surge observed in 2016 and 2020. The majority of the studies centered on surgical concerns. However, only a small proportion received financial support, which was particularly evident for funding originating from the private sector.

## Background

To keep up with the almost exponential progress in medical science, medical teaching must adapt to these advances. Alongside newly developed teaching methodologies, technological achievements offer the potential to align student education with the growing demands of clinical training [1].

Providing online educational materials represents the foundational form of digital teaching. Moreover, instructional videos—whether streamed lectures or on-demand content—have become a well-established format over the past few years [2]. Particularly during the recent Covid-19 pandemic, which led to necessary restrictions on in-person interactions, these formats have been increasingly integrated into student education [3].

While online courses and digital exams provide great benefits for acquiring and testing theoretical knowledge, learning practical skills and clinical competences requires different teaching methods. Using the advancements in Virtual Reality (VR) technology for training relevant clinical skills offers various advantages for students and patients [4] and is expected by medical students to be usefully integrated into medical curricula.

VR in general can be categorized into screen-based applications (e.g. in surgical simulators) and immersive solutions (involving a so called “head-mounted display”), which is considered even more useful in terms of knowledge acquisition [5].

The different medical disciplines comprise heterogeneous requirements for students. Internal medicine disciplines, for example, require a profound insight into the physiology of the organ systems and pathophysiology of the individual diseases. Surgical disciplines, on the other hand, require a wide range of anatomical and manual skills that can only partly be taken from a classical textbook. Here in particular, VR could offer innovative solutions for effective student teaching [6,7].

It is common sense that the number of publications in the field of VR in medical education has surged in parallel with the rapid advancements in technology over the past years. However, a comprehensive analysis and documentation of this phenomenon is currently lacking in the existing literature. By addressing this gap, our study aims to provide a bibliometric examination of the development in publications and thereby contributing to a deeper understanding of the evolution of the field and its scholarly pursuits. In the framework of this research, we want to test the hypothesis that subjects that are particularly dependent on the learning of hard skills (manual skills) and practical skills (such as examination techniques) benefit from VR in teaching and are therefore particularly involved in scientific research.

## Methods and study design

### Study design

**Fig. 1:**
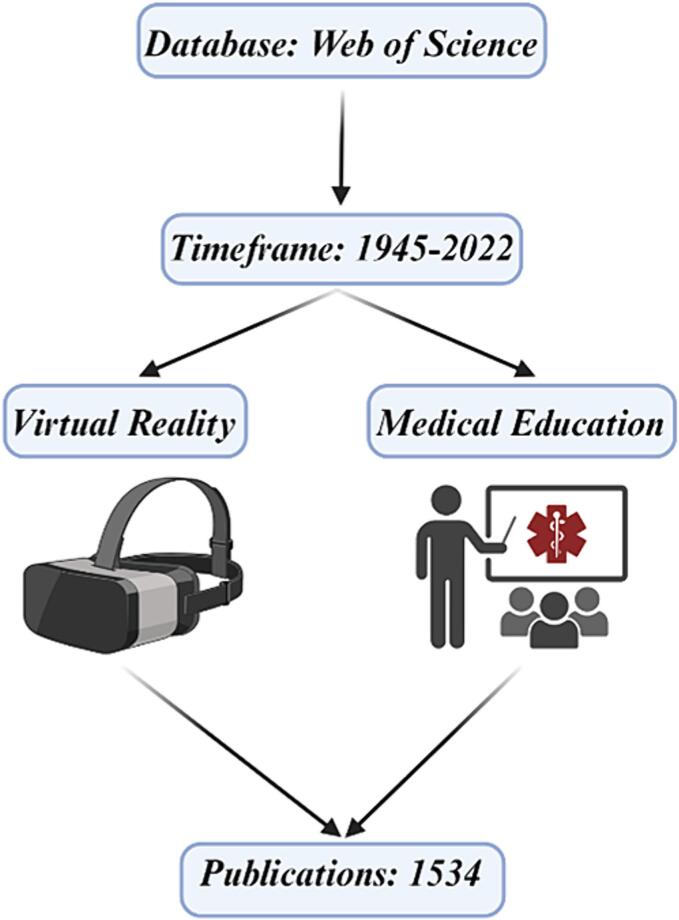
Study design.

In this study, the publication behaviour on the use of VR in medical education was investigated. All published studies from 1945 to 2022 (date of data collection 01.07.2023) were recorded, analysed, and scientifically compared with each other (Fig. 1). Special attention was given to the origin and funding of the publications as well as to the progress over time and the citation behaviour. Furthermore, it was explored which disciplines have produced most of the research. No approval from the institutional review board was required to conduct the study.

### Database and search strategy

Data collection was carried out using the worldwide established, multi-disciplinary search platform for bibliographic databases Web of Science™ (WoS). To include as many articles as possible on the topic of VR in medical education and at the same time to prevent the inclusion of non-topic-related publications, broad but specific search terms and matching Boolean operators were identified in accordance with the WoS guidelines resulting in the following queries: (TS = ((“virtual reality”) or (vr))) and (TS = ((medical education) or (medical teaching)). The search terms were selected based on clinical experience of the authors and search terms of previous publications. The observation period chosen was the beginning of the database (1945) to 2022.

### Analysis

The majority of the analysis was performed using the established analysis function of the WoS. The publications and citations per year were exported to an Excel table (Excel 2021, Microsoft Corporation, Redmond, Washington, USA) and further processed. The analysis of the different types of publications, as well as citation analysis (publications with most citations and number of citations per year) were based on the indication of the WoS.

The included studies were assigned to their scientific category by the WoS. For each publication, all cited references were provided with the respective subject areas assigned to the journals in which the cited references occur. Then - determined by the most frequent subject area - the publication was reclassified to the most frequently occurring category from this distribution. Furthermore, for the purpose of analysis, a distinction was made between clinical subject areas (e.g. orthopedics) and non-clinical subject areas (e.g. computer science). Assignment to a country was based on the location of the authors' institution.

Funding agencies were identified through the WoS when indicated by the authors. Agencies were then categorized as either private sector (e.g. corporations) or nonprivate sector (governmental, nonprofit, and nongovernmental organizations (NGOs)), depending on their economic background. This assignment was performed manually.

Statistical calculation and figures were performed and generated using Excel, Biorender (2023 BioRender, Toronto, Ontario), and GraphPad Prism (GraphPad Prism 9, GraphPad Software, Boston, Massachusetts).

## Results

### Timeline of publications and citations

The first publication on VR in medical education appeared in 1993. Since this first study, there has been a steady increase in publications per year (Fig. 1).

The most common publications were articles (1278 publications; 77.6 %), followed by review articles (320 publications; 19.4 %), proceedings papers (62 publications; 3.8 %), early access publications (35 publications; 2.1 %), meeting abstracts (17 publications; 1 %), book chapters (9 publications; 0.6 %) and letters (4 publications; 0.2 %).

**Fig. 2:**
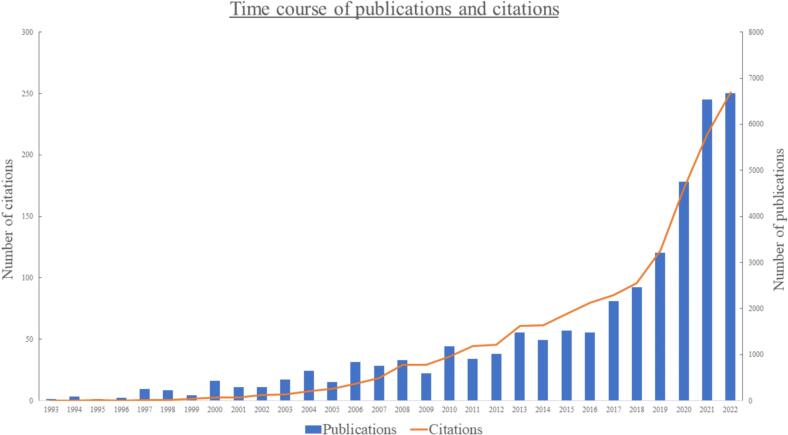
Timeline of publications and citations per year in VR in medical education. The left Y-axis represents the number of publications per year (blue bars), the right Y-axis the number of citations per year (orange line).

In total, the publications were cited 42,655 times. This resulted in an average of 27.64 citations per publication. As with the publications, the rate of citations per year has increased over the years, with an almost exponential increase in the last few years (Fig. 2). The publications with the most citations are displayed in Table 1.

**Table 1:** Top 5 most cited publications.

Publication	Citations
1. Issenberg SB, McGaghie WC, Petrusa ER, Lee Gordon D, Scalese RJ. Features and uses of high-fidelity medical simulations that lead to effective learning: a BEME systematic review. Med Teach. 2005 Jan;27(1):10–28.	2050
2. Reznick RK, MacRae H. Teaching surgical skills--changes in the wind. N Engl J Med. 2006 Dec 21;355(25):2664–9.	1160
3. Eysenbach G. Medicine 2.0: social networking, collaboration, participation, apomediation, and openness. J Med Internet Res. 2008 Aug 25;10(3):e22.	612
4. Wulf G, Shea C, Lewthwaite R. Motor skill learning and performance: a review of influential factors. Med Educ. 2010 Jan;44(1):75–84.	465
5. Jensen L, Konradsen F. A review of the use of virtual reality head-mounted displays in education and training. Educ Inf Technol 2018 Jul;23(4)1515–1529	*406*

### Participating disciplines

**Fig. 3:**
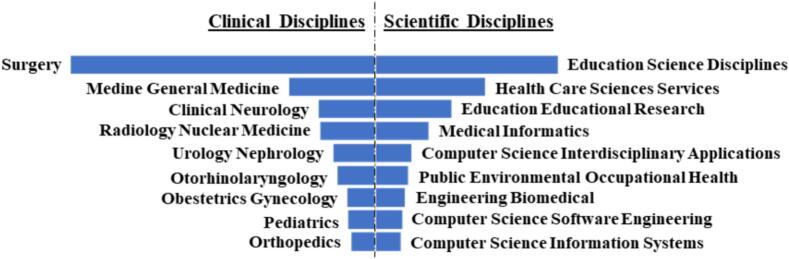
Ranking of the clinical and scientific disciplines most involved in the publication on the use of VR in medical education.

A variety of disciplines participated in research on the utility of VR in medical education and can be categorized in to clinical and non-clinical specialties. However, publications from surgical specialties led the overall and clinical ranking (415 publications, 25.20 %), followed by General Medicine Internal (117 publications. 7.1 %), Clinical Neurology (77 publications, 4.7 %, Radiology and Nuclear Medicine (75 publications, 4.6 %), Urology/Nephrology (57 publications, 3.5 %), Otorhinolaryngology (52 publications, 3.2 %), Obstetrics/Gynecology (38 publications, 2.3 %), Pediatrics (37 publications, 2.3 %) and Orthopedics (33 publications, 2 %) (Fig. 3).

The leading publications that deal with general aspects of teaching and not specifically assigned to a clinical specialty or technical aspects were divided as follows: Education Scientific Disciplines (279 publications, 16.9 %), Health Care Sciences Services (168 publications, 10.2 %), Education Educational Research (117 publications, 7.1 %), Medical Informatics (82 publications, 5 %), Computer Science Interdisciplinary Applications (55 publications, 3.3 %), Public Environmental Occupational Health (50 publications, 3 %), Engineering Biomedical (45 publications, 2.7 %), Computer Science Software Engineering (41 publications, 2.5 %) and Computer Science Information Systems (39 publications, 2.4 %) (Fig. 3).

### Countries

**Fig. 4:**
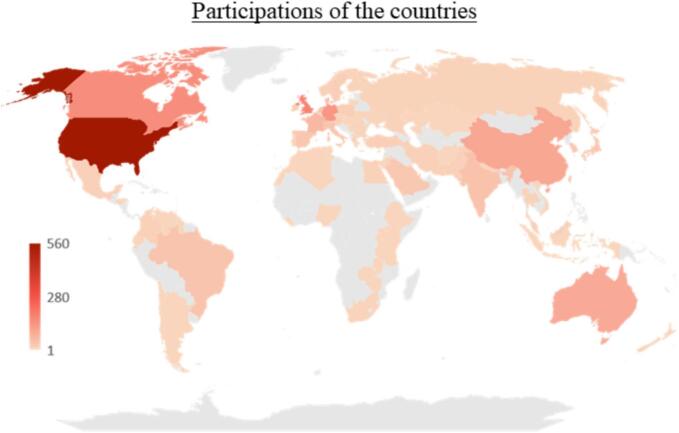
Heat map of the publications on VR in medical education per country. Countries in grey have no publications. The intensity of red correlates with the number of publications (transparent = few, intensive = many).

Authors from English-speaking countries contributed the most studies to the investigated research area (USA: 560 publications, 34 %; England: 179 publications, 10.9 %; Canada: 156 publications, 9.5 %). Germany and China ranked 4th and 5th in the international comparison (Germany: 139 publications, 8.4 %; China: 100 publications, 6 %). Fig. 4 depicts the total number of publications of the countries.

### Funding

Of all publications on VR in medical education 37.1 % of the publications stated that they had received funding. 831 different organizations were involved, which provided financial support 1,199 times. Among the 100 organizations with the highest number of fundings, only 8 private companies contributed to a total of 21 fundings (General Electric: 5; Johnson & Johnson: 3; Johnson & Johnson USA: 3; Boston Scientific: 2; Intuitive Surgical: 2; Medtronic: 2; Olympus: 2; Stryker: 2). The 10 most involved organizations are shown in Table 2.

**Table 2:** Leading funding agencies.

Funding agency	Number of fundings
1. United States Department Of Health Human Services	44
2. National Institutes Of Health	37
3. National Natural Science Foundation Of China	20
4. Ministry Of Science And Technology Taiwan	15
5. UK Research Innovation	14
6. German Research Foundation	13
7. Projekt Deal	13
8. National Institutes Of Health Research	11
9. European Commission	10
10. National Research Foundation Of Korea	9

## Discussion

This study investigates the research progress in the field of VR in medical education, adopting classical bibliometric methods. Attention was focused on differences among individual medical disciplines besides monitoring the temporal trends of publications, citations, authorship, and institutes at country level.

VR has initially been introduced in medical teaching research in 1993. The work of Kaltenborn E. was mainly focused on possible applications in medicine and already discussed medical teaching as a possible field of application [8]. While the number of publications has increased only moderately in the first few years, there has been a significant rise in publications from 2016 onwards. This trend continued until the end of the reviewed period in 2022. The surge in research, in addition to a general increase in research in most medical fields [9,10], can be explained by both the technical development in the field of VR and the increasing interest and awareness of innovative digital tools for medical education.

In 2014, Google LLC released their low-cost VR glasses, Google Cardboard, alongside high-end systems Samsung Gear VR (Samsung, 2015) and Oculus Rift (Meta Platforms, 2016). These glasses enabled an immersive experience, a milestone in the history of VR, despite the technical limitations of the previous generation. Scientifically and commercially, there has been a growing demand for immersive VR offerings [11].

Furthermore, there has been a second significant rise in publications from 2020 onwards. In addition to the more technical cause, this is most likely related to changes in teaching as a result of the pandemic. Conducting medical education while adhering to social distancing measures acted as a catalyst for digitization in many countries [12], resulting in the ability to attend lectures remotely without difficulty. Disciplines in which essential abilities are mostly acquired in practical courses encountered greater challenges. Advancements in solutions necessitate increased research in the corresponding sectors, which consequently led to a substantial upsurge in VR publications.

In addition to the technical and epidemiological rationales set forth, VR research is presently sought after for additional objectives. The implementation of VR in medical education could result in superior educational outcomes characterised by greater student self-efficacy, elevated student satisfaction levels and reduced student anxiety [13].

Our analysis revealed that surgical disciplines, particularly, are at the very forefront of VR research. Although bibliometrically examined for the first time within our study, the results match the students' anticipations [14]. Imposing immersive teaching landscapes through VR headsets enhances the understanding of essential surroundings and procedures. Specifically, technical skills like those needed in surgical fields can be trained with the utmost realism and accuracy. On the other hand, subjects that require more soft skills, such as decision-making and interpersonal skills, do not necessarily require high-tech teaching approaches, although the use of VR methods could also be beneficial in these areas [15]. This is supported by the inclusion of two publications [16,17] centered around the instruction of motor abilities via VR applications within the top 5 most cited publications.

In particular, the English-speaking countries United States, United Kingdom, and Canada, followed by Germany and China, accounted for the majority of VR research in medical education. Likewise, other studies [18] on the digitization of teaching revealed the same geographical distribution of the research landscape. Research, particularly in highly technical areas such as VR, can be very cost-intensive. Thus, only high-income countries are able to procure the required equipment, which can now be priced at up to £3499 per end-user device (RRP expected of the VR glasses Apple Vision Pro, Apple, California USA). In contradiction to the high costs is the comparatively low level of funding.

The use of VR depends largely on the development of the software used and is therefore diverse. The application in our clinic currently includes, for example, the development of a virtual environment that can visualize the orthopedic examination on the patient and a virtual suture trainer. In some countries, training on surgical simulators, such as a simulator for knee arthroscopy, is mandatory before applying what has been learnt on patients in the operating theatre. In the future, the establishment of VR simulators in surgical teaching can facilitate the learning of practical skills. In teaching, surgeons should open up to the new techniques and motivate their students and residents to continue their digital training with the new possibilities.

On average, only one out of three publications received a funding grant. This finding aligns with the observation that research in education is often underfunded [19]. A crucial factor may be the significantly low involvement of private companies in research. In contrast, private companies act as a vital driving force for the implementation of expensive research fields in other areas. It is clear that only highly skilled medical professionals will be able to make valuable contributions to the future advancement of technology marketed by companies, as this requires significant proficiency and expertise. However, the study's methodology prevents any conclusive statements about the extent of financial support. Hence, it is imperative that further research urgently investigates this matter to raise public awareness and highlight the need for additional financial support for research within the field of VR in medical education. In addition, bibliometric studies, are subject to specific limitations. While such studies can comprehensively analyze all publications on VR, it is not possible to draw conclusions about their content. This study's inclusion of only English-language publications from a single database may potentially result in the exclusion of important works. The use of external programs could mitigate this. However, the considerable quantity of bibliometric software currently available is not exempt from errors and may once again distort results. Additionally, the algorithm of the Web of Science made the discipline assignment, which could result in an inaccurate classification. Despite these limitations, this study constitutes the first exhaustive analysis of publications on the use of Virtual Reality in medical education.

## Conclusion

Since the first publication of research on VR in medical education in 1993, there has been a steady increase, driven in particular by technical and epidemiological factors. The field of surgery, with its practical requirements, has a significant research interest and consequently contributed the most publications. Overall, research in this specific area tends to be under-resourced, especially funded by private companies.

## Funding sources statement

The authors report that they received no funding for this article.

## Ethical approval statement

No approval from the institutional review board was required to conduct the study.

## CRediT authorship contribution statement

**M.A. Wolf:** Writing – original draft, Methodology, Formal analysis, Data curation, Conceptualization. **M. Mergen:** Writing – review & editing, Conceptualization. **P. Winter:** Writing – review & editing, Conceptualization. **S. Landgraeber:** Writing – review & editing. **P. Orth:** Writing – review & editing.

## Declaration of competing interest

None.
